# Marital status and risk of type 2 diabetes among middle-aged and elderly population: a systematic review and meta-analysis

**DOI:** 10.3389/fmed.2024.1485490

**Published:** 2025-01-03

**Authors:** Mohammad Amin Karimi, Sara Binaei, Seyed Hadi Hashemi, Pegah Refahi, Ensiyeh Olama, Elnaz Olama, Aydin Mohammadpour, Reza Mostafaei Yonjali, Mohadeseh Poudineh, Niloofar Deravi

**Affiliations:** ^1^School of Medicine, Shahid Beheshti University of Medical Sciences, Tehran, Iran; ^2^Endocrinology and Metabolism Research Center, Hormozgan University of Medical Sciences, Bandar Abbas, Iran; ^3^School of Medicine, Isfahan University of Medical Sciences, Isfahan, Iran; ^4^Student Research Committee, Faculty of Medicine, Mashhad University of Medical Sciences, Mashhad, Iran; ^5^Faculty of Medicine, Georgian National University SEU, Tbilisi, Georgia; ^6^Student Research Committee, Tabriz University of Medical Sciences, Tabriz, Iran; ^7^Student Research Committee, School of Medicine, Zanjan University of Medical Sciences, Zanjan, Iran; ^8^Student Research Committee, School of Medicine, Shahid Beheshti University of Medical Sciences, Tehran, Iran

**Keywords:** diabetes, divorced, married, relationship, single, widow, T2DM, meta-analysis

## Abstract

**Background:**

Marital status is among the factors influencing type 2 diabetes mellitus (T2DM). However, the precise relationship remains incompletely understood. This meta-analysis aims to evaluate the association between marital status and the incidence of T2DM.

**Methods:**

A review and meta-analysis of observational studies were conducted to investigate the relationship between marital status and diabetes incidence. We searched three databases, including PubMed, Google Scholar, and Scopus, for relevant studies published up to August 16th, 2023. In our initial search, we identified a total of 358 articles. After a demanding screening process involving evaluating titles, abstracts, and full-text content, we ultimately included six studies for our meta-analysis.

**Result:**

Comprising a total of 1,440,904 participants, our study found that in comparison to married individuals, unmarried participants exhibited a higher likelihood of developing diabetes [odds ratio (OR): 1.47, 95% confidence interval (CI): 0.88–2.45, *I*^2^: 91%, *p*-value = 0.14]. Divorced participants had a reduced likelihood of developing diabetes compared to married participants (OR: 0.84, 95% CI: 0.77–0.91, *I*^2^: 17%, *p* < 0.001). Similarly, widowed participants showed a lower risk of developing diabetes compared to divorced participants (OR: 0.35, 95% CI: 0.26–0.46, *I*^2^: 83%, *p* < 0.00001).

**Conclusion:**

This study provides strong evidence of links between marital status and type 2 diabetes risk. Unmarried individuals are more susceptible to T2DM, divorced individuals have a lower risk, and widowed individuals exhibit reduced T2DM risk. Further research should investigate underlying mechanisms and confounding factors.

## Introduction

One of the world’s most prevalent chronic diseases is diabetes mellitus (DM), which ranks among the top 10 causes of mortality in the USA (1). Based on the World Health Organization (WHO) definition, DM is characterized by enduring metabolic dysfunction, leading to elevated blood glucose levels and adverse impacts on various bodily organs such as the cardiovascular, eyes, kidneys, and nervous systems. Most patients diagnosed with diabetes mellitus present as type 2 diabetes mellitus (T2DM). This condition is developed by insufficient insulin secretion from the pancreatic cells, tissue insensitivity to insulin, and limited compensatory insulin release (2–4). The increasing prevalence of T2DM can be attributed to lifestyle changes and overall health status improvements. Studies showed that nearly 300 million individuals could be affected by this condition by 2025 (5).

Moreover, it has been proved that diabetes was responsible for nearly 4 million fatalities among individuals aged 20 to 79 in 2019, accounting for roughly 11% of the total global mortality. Furthermore, more than 46% or about 2 million of these deaths occurred in individuals under 60 (6). Additionally, T2DM has been definitively linked to heightened risks of cardiovascular disease (CVD) (7), renal dysfunction (8), and increased mortality rates resulting from infections (9), as well as an elevated susceptibility to certain cancers, including pancreatic, hepatic, renal, thyroid, breast, and uterine corpus cancers (10).

One of the most important psychological factors influencing many aspects of a person’s health state is their marital relationship and its quality. The word “marital relationship” refers to a system for evaluating the overall marriage quality using positive and negative traits (11). Numerous studies have established that marital status significantly influences health outcomes, including the development and progression of chronic diseases such as cancer (12, 13), hypertension (14), and cardiovascular diseases (15). The relationship between marital status with a particular spotlight on T2DM has been the main point of multiple investigations (16–20). Specific studies have illuminated constructive consequences concomitant with marital status (21, 22). Notably, research among males has spotlighted the correlation between suboptimal marital quality, solitary marital status, and enhanced susceptibility to T2DM, especially among widowers (16). Furthermore, marital status has been identified as an independent risk factor for T2DM within the male demographic. Conversely, widowhood has been associated with a decreased T2DM risk in the female population (23). Another study showed that spousal participation is very important in improving teamwork in diabetes management and leads to better patient blood glucose control (24).

Consequently, due to heterogeneity and disagreement among the results of earlier studies, the effect of marital status on any component of T2DM and associated health effects continues to be a challenging issue despite the vast number of studies conducted. To the best of our knowledge, this study is the first to examine the relationship between T2DM and each marital status, including married, never married, widowed, and divorced.

## Methods

### Protocol and registration

This study was conducted in strict accordance with the Preferred Reporting Items for Systematic Reviews and Meta-Analyses (PRISMA) guidelines, ensuring methodological rigor and transparency. The study protocol has been meticulously developed and registered with the Open Science Framework (OSF), available at https://osf.io/a86vp

### Search strategy

This meta-analysis searched three international databases: PubMed, Scopus, and Google Scholar. The search was performed in these databases from the beginning to August 16th, 2023, without any limitations or filters. The search terms consisted of the terms “diabetes OR diabetes mellitus” AND “marital status OR marital OR married OR marriage OR spouse OR widow OR widowed divorced” and MeSH terms (for PubMed). Supplementary Table S1 contains information on the search queries and the number of records in each database.

### Screening and inclusion and exclusion criteria

We employed EndNote software to conduct the initial screening of records sourced from multiple databases. To ensure data integrity, duplicate database entries were eliminated utilizing the “Find Duplicates” function in EndNote. Subsequently, studies unrelated to marital status and those lacking diabetic populations were excluded through a meticulous screening of titles and abstracts. The full texts of the studies identified in the prior phase underwent a comprehensive evaluation to determine the final set of studies meeting the pre-defined inclusion and exclusion criteria for incorporation into this review.

We included observational studies (cross-sectional, cohort, case reports, and case series) that met the following inclusion criteria: observational studies on the correlation between marital status and the susceptibility to T2DM. The exclusion criteria encompassed review papers, editorials, comments, *in vivo* and *in vitro* investigations, and randomized clinical trial studies. In addition, several pertinent research were identified by manually examining the references cited in the articles from the original search.

### Quality assessment

The Newcastle–Ottawa Scale (NOS) for non-randomized studies was used to assess the risk of bias in included studies (25). NOS evaluates studies according to pre-specified items, including selection, comparability, and exposure. Studies with a total NOS ≥8 are considered high quality.

### Study selection and data extraction

After removing duplicate entries, two reviewers (MAK and SH) independently examined each title and abstract. Disagreements were resolved by utilizing a third reviewer or achieving a consensus (SB). The studies that met the inclusion criteria were obtained in their entirety and underwent an independent analysis by two authors (MAK and ElO). When the reviewers could not reach a consensus, a third author (RY) was consulted. Studies that did not meet the specified criteria for inclusion were finally excluded.

Two reviewers (MAK and ElO) independently extracted the following data from the included papers using a pre-existing standardized template: author and publication year, nation, study design, follow-up period, population and gender, age, and marital status. In instances of disagreement between reviewers, a third author (RY) was consulted.

### Statistical analysis

We employed the STATA 13.1 software, developed by StataCorp LP in College Station, TX, United States, to conduct our data analysis. The findings were reported as combined odds ratios (ORs) with a 95% confidence interval (CIs) and shown in a forest plot. In order to measure the differences between the studies that met the criteria, we employed the *I*^2^ statistic (26) and applied the random effects model when we found substantial variation (*I*^2^ is greater than 50%) (27). Following a thorough examination of the symmetry of the funnel plot and Egger’s regression analysis, we explored the possibility of publication bias (28).

## Results

### Study selection

**Figure 1:**
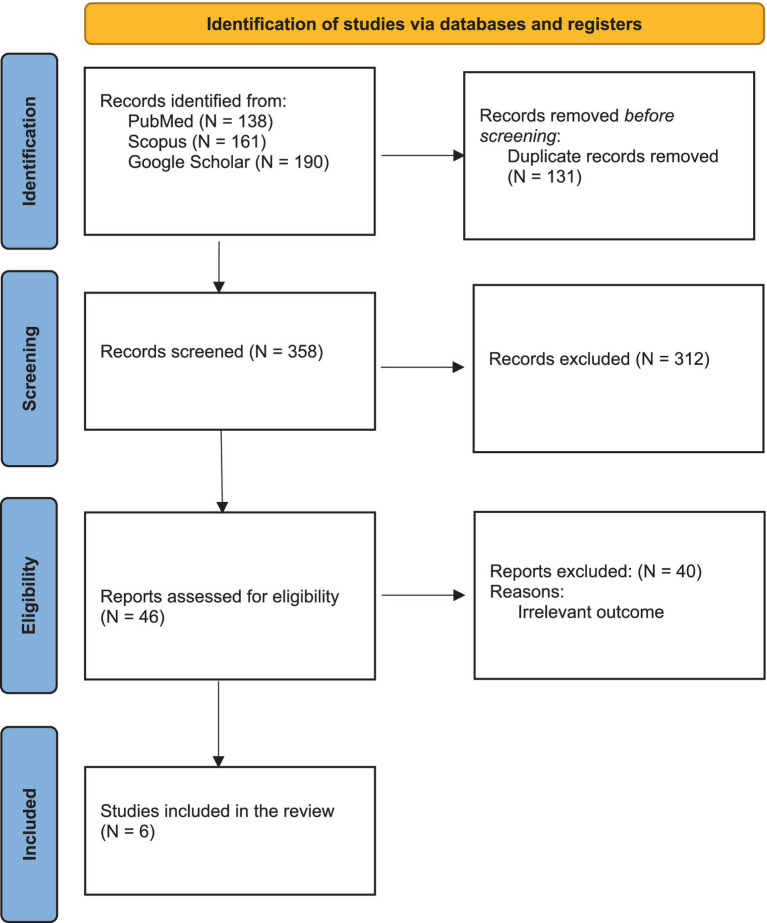
PRISMA flow diagram outlining the systematic review process. The figure illustrates the identification, screening, eligibility, and inclusion stages of the systematic review. A total of 489 records were initially identified from three databases: PubMed (*n* = 138), Scopus (*n* = 161), and Google Scholar (*n* = 190). After removing duplicates (*n* = 131), 358 unique records were screened based on titles and abstracts, excluding 312 irrelevant studies. Full-text assessments were conducted on 46 reports, with 40 excluded due to irrelevant outcomes. Ultimately, six studies meeting the inclusion criteria were selected for the final review and meta-analysis.

A total of 489 studies were discovered for this analysis by conducting a primary literature search in Scopus, PubMed, and Google Scholar. Three hundred fifty-eight studies remained after removing the duplicates. Out of the total, 312 cases were deemed irrelevant to the study’s objective and were consequently removed through title/abstract screening. Afterward, a total of 46 records that could potentially be relevant were carefully examined in a full-text review. Out of them, 40 cases were also excluded due to the presence of irrelevant data. The selection and screening process for our study, encompassing identification, eligibility assessment, and final inclusion of studies, is comprehensively depicted in Figure 1.

### Baseline characteristics

Finally, six articles with a total population of 1,440,904 were reviewed. Five of these six observational were cohort research (23, 29–32), and one was cross-sectional (33). These examinations were performed in Iran (23, 33), Brazil (29), the USA (30, 31), and Finland (32). The average age of those involved in these studies varied from 41 to 77 years. The follow-up duration of cohort studies ranged from 1 to 22 years. The years of investigation were 2002 through 2023. In each of the six incorporated studies, all reported the prevalence of marriage among individuals diagnosed with diabetes (23, 29–33), two out of the six studies addressed the status of being unmarried in diabetic patients (29, 32), while five out of the six studies examined the prevalence among widowed or divorced individuals within the diabetic population (23, 30–33). Additionally, four of the six studies investigated the occurrence of diabetes among individuals categorized as single or never married (23, 30, 31, 33).

### Married vs. unmarried

**Figure 2:**
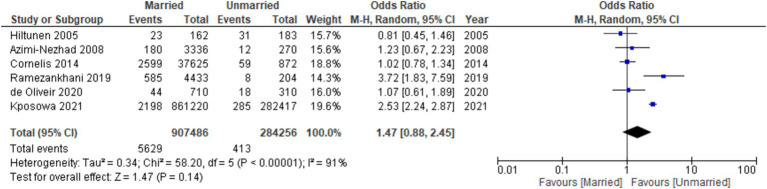
Forest plots of diabetes risk in married vs. unmarried.

**Figure 3:**
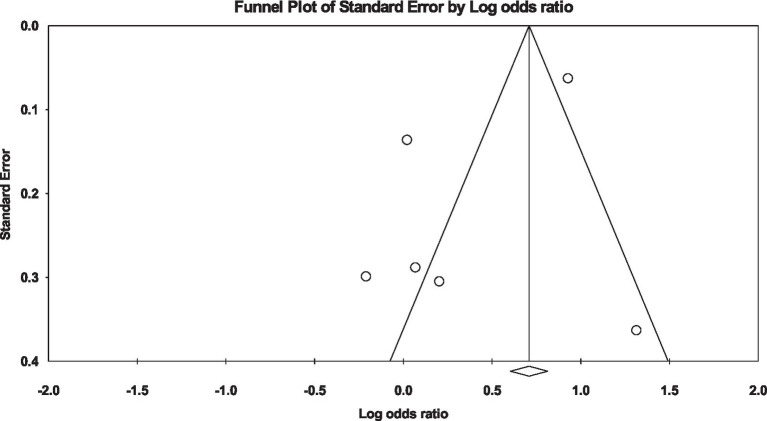
Funnel plot of diabetes risk in married vs. unmarried.

In the conducted analysis, it was observed that unmarried participants exhibited a higher propensity for developing diabetes in comparison to their married counterparts (OR: 1.47, 95% CI: 0.88 to 2.45, *I*^2^: 91%, *p* = 0.14) (Figure 2). Additionally, in evaluating publication bias, the funnel plots that were conducted displayed no discernible asymmetry in the distribution pattern of the studies (Figure 3).

### Married vs. divorced

**Figure 4:**
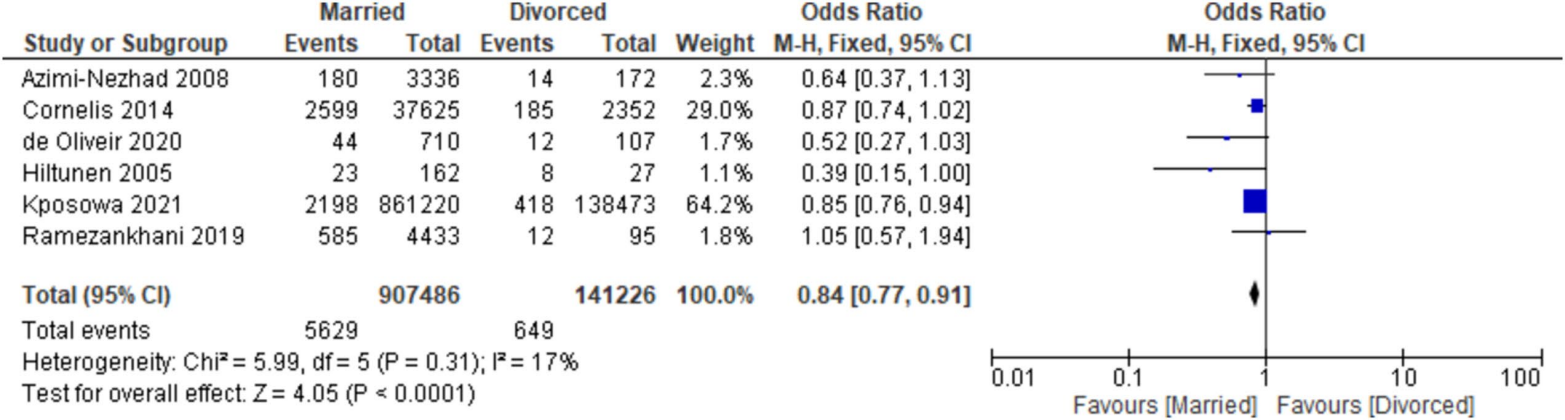
Forest plot of diabetes risk in married vs. divorced.

**Figure 5:**
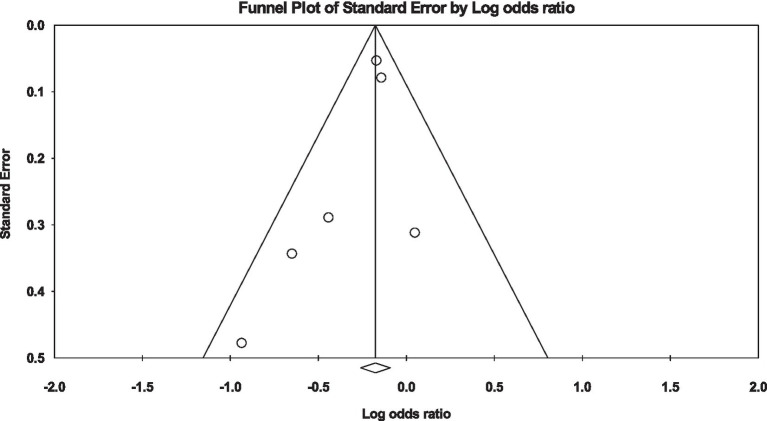
Funnel plot of diabetes risk in married vs. divorced.

The analysis revealed noteworthy findings in the context of marital status and the risk of developing diabetes. When comparing divorced individuals to their married counterparts, a lower likelihood of diabetes development was observed among the divorced group [OR: 0.84, 95% CI (0.77, 0.91), *I*^2^: 17%, *p* < 0.001] (Figure 4). Assessment for publication bias through funnel plots indicated no significant asymmetry in the distribution pattern of studies (Figure 5).

### Married vs. widowed

**Figure 6:**
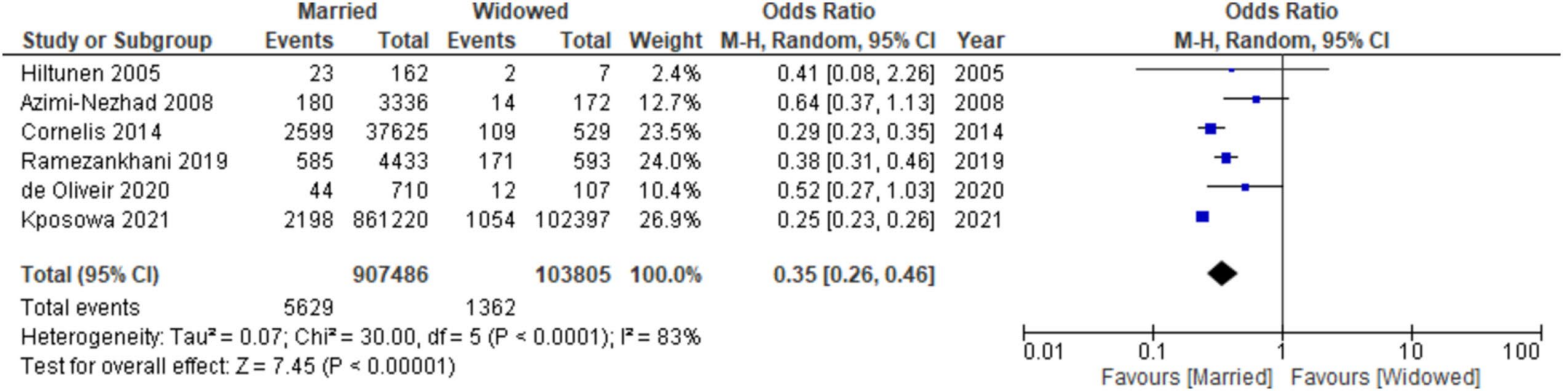
Forest plot of diabetes risk in married vs. widowed.

**Figure 7:**
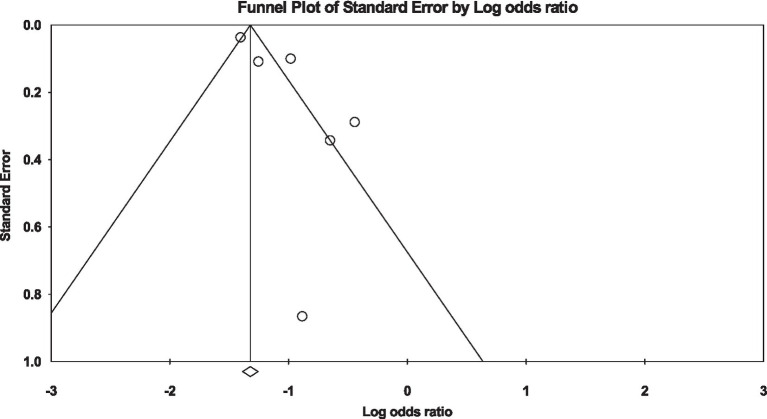
Funnel plot of diabetes risk in married vs. widowed.

Similarly, when evaluating the risk in widowed individuals compared to divorced individuals, a significantly lower likelihood of developing diabetes was evident among the widowed participants [OR: 0.35, 95% CI (0.26, 0.46), *I*^2^: 83%, *p* < 0.00001] (Figure 6). Furthermore, examination via funnel plots did not exhibit any substantial asymmetry in study distribution, signifying no evident publication bias (Figure 7).

## Discussion

This systematic review and meta-analysis aimed to examine the relationship between marital status and the risk of T2DM. Our objective was to determine whether marital status significantly affects an individual’s susceptibility to type 2 diabetes, filling a critical void in the existing literature. The present study comprehensively analyzed six eligible research, encompassing 1,440,904 participants. The findings of this analysis have revealed that unmarried individuals exhibited a higher propensity for the development of diabetes in comparison to their married counterparts. Furthermore, divorced participants demonstrated a lower likelihood of developing diabetes when contrasted with their married counterparts. Similarly, widowed participants also displayed a reduced likelihood of diabetes development compared to their married counterparts (see Table 1).

**Table 1:** Detailed summary of the extracted data from the included studies.

Author	Year	Country	Study design	Follow up duration	Number of participants	Marital status	Mean age	Sex (female)
Ramezankhani et al. (23)	2019	Iran	Cohort	More than 12 years	9,737 Iranian adults	Men: Never married: 213 Married: 4,153 Widowed/divorced: 46 Women: Never married: 204 Married: 4,433 Widowed: 593 Divorced: 95	47.6 ± 12.7	55%
Azimi-Nezhad et al. (33)	2008	Iran	Cross-sectional	Not applicable	3,778 aged ≥ 15 selected from the general population	Married: 3,073 Single: 255 Widowed/divorced: 150	Not mentioned	50.9%
de Oliveira et al. (29)	2020	Brazil	Cohort	Five years	1,125 individuals aged ≥ 18 (excluding diabetic patients)	Married: 706 Single: 310 Divorced/Widower: 100	Female: 41 ± 15 Male: 43 ± 17	57%
Cornelis et al. (31)	2014	USA	Cohort	22 years	41,378 men who were free of T2DM in 1986	Married: 37,625 Divorced/separated: 2,352 Widowed: 529 Never married: 872	Married: 53.2 ± 9.5 Divorced/separated: 50.2 ± 8.2 Widowed: 62.0 ± 8.6 Never married: 50.1 ± 9.2	0%
Hiltunen (32)	2005	Finland	Cohort	Between 1 September 1991 and 29 February 1992	379 individuals aged ≥ 70 selected from the general population	Married: 15 Unmarried: 30 Widowed: 17 Divorced: 29	Men: 75.7 ± 4.9 Women: 76.8 ± 5.0	63%
Kposowa et al. (30)	2021	USA	Cross-sectional	Not applicable	1,384,507 individuals aged ≥ 18 selected from the general population	Married: 861,220 Divorce/separated: 138,473 Single/never married: 282,417 Widowed: 102,397	Not mentioned	53%

Numerous studies have extensively explored the association between T2DM and marital status. The precise mechanisms contributing to the influence of marital status on T2DM remain incompletely understood. Previous research has proposed various hypotheses for these effects, encompassing psychopathological aspects, neuroendocrine pathways, health behaviors (including physical activity, dietary habits, and adherence), biological mediators, incorporating social cognitive processes, mental health outcomes, and immunological pathways (16, 34–36). Also, two principal theories have elucidated the health benefits attributed to marriage. The initial one relates to the “selection”: individuals in better health are more likely to enter into and maintain marriages. The second hypothesis reflects the post-marriage effect: tension reduction and adoption of healthful behaviors (37–39).

We have found that unmarried individuals were more likely to develop diabetes than married individuals [OR: 1.47, 95% CI (0.88, 2.45), *I*^2^: 91%, *p* = 0.14]. Cornelis et al. (31) revealed that unmarried males exhibited a 16% elevated susceptibility to developing T2DM compared to their married counterparts. They conducted a prospective analysis on male health professionals to investigate the probability of developing incident T2DM concerning their present marital status. Following a 22-year period of follow-up, it was determined that there exists a notable elevation in the likelihood of developing T2DM among males who are not married. Our findings are in agreement with Hiltunen’s (32) results, which showed that the prevalence of diabetes was lower among individuals who were married, in contrast to those who were unmarried. However, Azimi-Nezhad et al. (33) discovered that the prevalence of T2DM does not differ significantly between married and unmarried individuals.

Our analysis has shown that divorced participants were less likely to develop diabetes than married participants [OR: 0.84, 95% CI (0.77, 0.91), *I*^2^: 17%, *p* < 0.001]. Our finding is in contrast to the results of de Oliveira et al. (29) results. Their research findings showed a significant increase in body weight in individuals who stayed married or got married during the 5-year follow-up period. However, individuals who maintained their marital status despite experiencing substantial weight gain exhibited a notably reduced risk of developing diabetes compared to those who had gone through a divorce. Furthermore, Kposowa et al. (30) conducted a study revealing that men who had experienced divorce or separation had an increased risk of mortality due to diabetes mellitus. On the contrary, findings from Cornelis et al. (31) indicated no statistically significant elevation in the risk of M among men who were divorced, separated, or never married compared to their married counterparts. Also, Azimi-Nezhad’s et al. (33) investigation demonstrated that the prevalence of T2DM did not exhibit a significant difference between married, unmarried, bereaved, or divorced individuals.

Several studies have shown that those who are unmarried, divorced, or widowed may have negative health consequences. A survey conducted in Brazil showed that the incidence of diabetes was 60% higher among those who were widowed than those who were married (40). Our findings show that widowed participants were also less likely to develop diabetes compared to married participants [OR: 0.35, 95% CI (0.26, 0.46), *I*^2^: 83%, *p* < 0.00001]. These findings are in accordance with the research conducted by Ramezankhani et al. (23). The study revealed a substantial correlation between widowhood and a 31% diminished susceptibility to the development of T2DM in the female population. The results of this investigation further elucidated that, spanning 12 years, widowed women exhibited a reduced likelihood of developing Type T2DM compared to their married counterparts. Importantly, this association retained its statistical significance even after meticulous adjustment for confounding variables. However, these findings are incongruent with the results obtained by Cornelis et al. (31) and Kposowa et al. (30); individuals who have experienced the loss of a spouse are more susceptible to developing diabetes mellitus.

This systematic review and meta-analysis represents the first comprehensive investigation into the association between all major marital statuses—married, unmarried, divorced, and widowed—and the risk of T2DM, drawing on data from high-quality studies with a combined sample size exceeding 1.4 million participants. In contrast to prior studies, such as Leong et al. (41), which examined spousal diabetes and its relationship with shared socio-environmental factors, or Nikolic Turnic et al. (42), which assessed marital status in relation to obesity as an indirect risk factor for T2DM, this meta-analysis focuses directly on T2DM as the primary outcome. Furthermore, it differs from Ramezankhani et al. (23), which included T2D as part of a broader analysis of cardiovascular and metabolic outcomes but did not provide an in-depth examination of this condition. By incorporating a wide range of marital statuses and addressing underexplored categories such as divorced and widowed individuals, this study resolves inconsistencies in previous research and provides rough insights into marital status as a determinant of T2DM risk. These results contribute significantly to diabetes prevention research by addressing a previously overlooked factor and advocating for targeted public health interventions tailored to vulnerable marital groups.

Nonetheless, this research has limitations. The included studies exhibited considerable heterogeneity, and the limited availability of investigations in this field further restricts the generalizability of the findings. As this study is based on an observational design, it is limited in its ability to establish causal relationships between marital status and T2DM risk, as is typical of descriptive studies that rely on associations rather than direct cause-effect mechanisms. Additionally, demographic factors such as age, race, and participants’ backgrounds were not accounted for in the analysis, which might influence the observed associations. Future research is necessary to address these gaps and refine our understanding of the intricate relationships between marital status and T2DM risk. Despite these limitations, this study underscores the importance of considering marital status as a determinant of T2DM and lays the groundwork for future research and targeted public health strategies.

## Conclusion

This study reveals consistent associations between marital status and T2DM risk, with unmarried individuals displaying a higher susceptibility to T2DM, divorced individuals having a lower likelihood, and widowed individuals exhibiting reduced T2DM risk. These findings underscore the importance of considering marital status as a potential factor in assessing T2DM risk. However, this study has some drawbacks, such as the heterogeneity and the shortage of studies. To better understand the association between marital status and T2DM, future research should look into the underlying mechanisms and consider potential confounding factors.

## Data Availability

The original contributions presented in the study are included in the article/Supplementary material, further inquiries can be directed to the corresponding authors.
