# Supplementary material for: Marital status and risk of type 2 diabetes among middle-aged and elderly population: a systematic review and meta-analysis
Source: Front Med (Lausanne). 2025 Jan 3;11:1485490. doi: 10.3389/fmed.2024.1485490 (PMC11739031; doi:10.3389/fmed.2024.1485490)
Supplement: Supplementary file 1 [file Table_1.docx]

**Table S1: Search strategy**

| **Search engine** | **Search strategy** | **Time** | **Results** |
| --- | --- | --- | --- |
| PubMed | (((diabete*[Title]) OR ("diabetes mellitus"[Title])) AND (("marital status"[Title]) OR (marital [Title]) OR (marriage[Title]) OR (spouse*[Title]) OR (widow*[Title]) OR (divorce*[Title]) OR (married[Title]))) | August 16^th^,2023 | 138 |
| Scopus | ( ( TITLE (diabete* ) OR TITLE ("diabetes mellitus") ) ) AND ( ( TITLE ("marital status") OR TITLE ( marital ) OR TITLE ( married ) OR TITLE ( marriage ) OR TITLE ( divorce* ) OR TITLE ( spouse* ) OR TITLE ( widow* ) ) ) | August 16^th^,2023 | 161 |
